# Shear Capacity and Behaviour of Bending Reinforced Concrete Beams Made of Steel Fibre-Reinforced Waste Sand Concrete

**DOI:** 10.3390/ma14112996

**Published:** 2021-06-01

**Authors:** Marek Lehmann, Wiesława Głodkowska

**Affiliations:** Faculty of Civil Engineering, Environmental and Geodetic Sciences, Koszalin University of Technology, Sniadeckich 2, 75-453 Koszalin, Poland; wieslawa.glodkowska@tu.koszalin.pl

**Keywords:** steel fibre-reinforced concrete, steel fibres, waste sand properties, reinforced beam, shear cracking, shear capacity

## Abstract

Inthis paper, we report the results of our research on reinforced concrete beams made of fine aggregate fibre composite, with the addition of steel fibres at 1.2% of the composite volume. The fine aggregate fibre composite is a novel construction material, in which the aggregate used is a post-production waste. Twenty reinforced concrete beams with varying degree of shear reinforcement, in the form of stirrups with and without the addition of steel fibres, tested under loading. The shear capacity results of reinforced concrete beams made of the fine aggregate fibre composite being bent by a transversal force, as well as the cracking forces causing the appearance of the first diagonal crack, are discussed. The stages of functioning of such elements are described. Furthermore, the effect of the steel fibres on the reduction of diagonal cracking is analysed. Computation of the shear capacity of the tested elements is performed, based on the Model Code 2010 and RILEM TC-162 TDF standards, for two variants of the compression strut inclination angle θ that measured during testing, and the minimum(in accordance with the Model Code 2010 standard). We found that the SMCFT method part of Model Code 2010 showed the best compatibility with the experimental results. The tests and analyses performed demonstrate that the developed novel fibrecomposite—the properties of which are close to, or better than, those of the ordinary concrete—can be used successfully for the manufacturing of construction elements in the shear capacity aspect. The developed fine aggregate fibrecomposite could serve, in some applications, as an alternative to ordinary concrete.

## 1. Introduction

Concrete belongs to the class of brittle and barely deformable materials; therefore, it is susceptible to scratching and cracking. The addition of, for example, steel fibres to such materials allows us to obtain higher plasticity and cracking resistance properties. Fibre-reinforced concrete was first used 140 years ago when, in 1874, Bernard submitted his first patent application for steel fibre-reinforced concrete. Since that time, attempts have been made to both evaluate the impact of the fibres on concrete properties [1,2,3,4,5,6,7,8,9], as well as distribution of fibres in the concrete [10,11,12]. Fibre-reinforced concrete has, therefore, become an alternative to ordinary concrete.

The fibre-reinforced concrete filling, just as in the case of ordinary concrete, is a fine and coarse aggregate selected based on a continuous grading curve. Aggregate deposits occur in the Pomeranian area in Poland, in the form of a mixture of fine and coarse aggregates. The high demand for coarse aggregate has contributed to the development of a technique of its sourcing by washing it out from its deposits. This technique is called hydroclassification. The application of hydroclassification of natural aggregates results in the build-up of dumps of washed-out sand, from which coarse aggregate fractions have been eliminated. Such created excavations must be subjected to costly reclamation operations. An alternative for reclaiming these excavations is through the possibility to use waste sand as a valuable construction material. Due to the shortage of coarse aggregate in the north region of Poland, a concrete composite has been developed [11,13,14],included in which is the fine aggregate (i.e., waste sand). In the analysed fine aggregate cement composite, the coarse aggregate was substituted with steel fibres, in order to create steel fibre-reinforced waste sand concrete (SFRWSC).

The subject matter of our analysis is, therefore, the novel steel fibre-reinforced waste sand concrete. This novel fibre composite material, featuring high compression and tensile strength, was designed based on a fine post-production waste aggregate. The research work described in [12,14,15] stated that a fibre composite based on waste sand containing 1–1.5% steel fibres has the best physicochemical properties. If the steel fibre content exceeds 1.5%, some tested material features decrease or improve insignificantly. Therefore, the addition of steel fibres in excess of 1.5% makes the resulting concrete hardly usable, due to material workability, its mechano-physical properties, and cost. A steel fibre content equal to 1.2% seems to be the best possible option, considering the fundamental properties that the structural composite should have. The residual strength values of SFRWSC with a steel fibre content of 1.2%, defined in the EN 14651 standard [16], have been classified in accordance with Model Code 2010 [17] as class 7b, which means that this material is suitable for the manufacturing of structural elements, and traditional reinforcement may be reduced. A feature distinguishing the analysed SFRWSC is its ability to resist higher shear forces, compared to concrete without fibre reinforcement. An increase in the shear capacity may lead to a reduction of traditional shear reinforcement or the complete abandonment of such reinforcement, due to shear capacity at small loads.

Analysing the state-of the-art in the field of shear fibre concrete elements, the first works were started in the 1970s. Batson, in [18], published the results of research on the influence of the shape, quantity, and dimensions of fibre reinforcement on shear force. Based on these studies, he concluded that the stirrups can be replaced by round, flat, or crimped steel fibres, which effectively influence the shear capacity of the support areas. In 1986, Sharma [19] conducted research and confirmed the beneficial co-operation of fibres and stirrups meanwhile, in 1987, Narayanan and Darwish published a study [20] considering beams with crimped fibre content, different degrees of main reinforcement, and varying *a*/*d* ratio. The shear problem in fibre-reinforced elements is still relevant and remains of experimental research. You et al., in their work [21], presented the results of an experiment carried out on rectangular beams, considering the use of fibre reinforcement with and without stirrups. Their research showed that the shear load capacity increases significantly with increasing fibre content, and the addition of an appropriate percentage of fibres can change the failure mode from brittle failure at shear to a ductile mechanism. The stirrups can be partially replaced by steel fibres and the combination of steel fibres and stirrups showed a positive effect on the mechanical behaviour of the composite. Similar conclusions have been reached by Ding et al. [22] and Li et al. [6]. Zhao [23], in his work, additionally characterized the effect of fibres on the reduction of diagonal cracks and strain after cracking. Analyses of the level of the scale effect by fibres in the shear load capacity [24]. Reviews of the state of knowledge of fibre-reinforced shear elements have been presented in the manuscripts [25,26], among others. Although the shear issue has been dealt with in numerous papers, there is still a need for further research, in order to analyse this issue in a more insightful way.

Knowledge of the bahavior and failure of reinforced concrete structures is of great technical and economic importance. Experimental testing of such components is laborious and costly. Therefore, the possibilities of numerically analyzing the work of reinforced concrete elements are often used, e.g., with the help of the Finite Element Method (FEM). In addition, along with the development of new materials and research methods, intensive scientific work is carried out on the use of numerical methods for modeling physical processes, development of material damage to destruction, modeling of ultra-high performance concrete, reinforced concrete shells and walls or structure strengthening [27,28,29,30]. The problem of shear in reinforced concrete beams [31,32] and in fiber-reinforced beams [33,34,35] is also subjected to numerical modeling. Talavera-Sanchez et al. [33] presented the test results of 16 beams with various parameters, including steel or macro-synthetic fibers, the presence or absence of transverse reinforcement, different shear-to-depth ratios, and different transverse reinforcement values. In numerical modeling was used the nonlinear finite element analysis following the smeared crack approach and a total strain-based crack material model. Numerical modeling has shown that the nonlinear finite element model can predict the behavior and strength of beams with transverse reinforcement with high accuracy. For members without transverse reinforcement, the shear capacity is acceptable, but some doubts remain unclear as these beams have large critical diagonal crack failure. In the manuscript of Amin and Foster [34] comparison of full scale SFRC beams ATENA 2D smeared crack models were described. The ATENA 2D integrated with a constitutive law derived after an inverse analysis from prism bending tests. The numerical model is validated against experimental results obtained. Authors analyzed experimental and numerical shear strength, deflection and diagonal crack pattern. It was shown that numerical model compared well with the experimental data in capturing the linear and non-linear responses of the beams. Some studies also concerned beam sections other than rectangular ones, and Baross and his team [35] dealt with modeling of T-sections. The studies of fiber-reinforced concrete T-beams damaged due to shear were analyzed in comparison to various numerical models, i.e., smeared crack model, discrete crack model, concrete damage plasticity model, and lattice discrete particle model. The models were analyzed in terms of deflection, strain and diagonal cracking. The authors obtained results with different accuracy in relation to the experimental results, stating that for the lattice discrete particle model, the best means of agreement are obtained.

The research work published in [12,15,36] concluded that SFRWSC without steel fibres behaves like ordinary concrete in bent elements provided with conventional reinforcement. The addition of steel fibres considerably improves the flexural capacity of such elements, thanks to which, the reduction of conventional reinforcement due to bending moment is possible. The addition of steel fibres also limits the width of cracks perpendicular to the element axis. These properties provide a possibility to use this material in the production of structural elements such as flooring slabs, beams, or coatings. Thanks to its mechano-physical properties, SFRWSC may, in some cases, serve as a substitute for ordinary concrete.

Considering the abovementioned achievements to date, the objective of the research presented in this paper was to show that the novel SFRWSC, with 1.2% fibre content, could be used for the production of reinforced concrete elements that are subject to bending functioning under shear force. To date, no research work has been performed on reinforced concrete elements made of SFRWSC that are subjected to bending by a transversal force. Therefore, an assumption was made, namely that steel fibres used as reinforcement can contribute to an improvement of the shear capacity of such elements. Another objective is to prove that, by using steel fibres, conventional shear reinforcement can be reduced, as well as reducing the diagonal crack width.

The design of fibre-reinforced concrete cross-sections functioning under shear force is still an unexplored issue [37,38]. The first design methods were based on experimental tests and had a limited scope of application. After the publication of two European standards—RILEM -TC-162-TDF [39] and Model Code 2010 [17]—the dimensioning of fibre-reinforced concrete cross-sections functioning under shear force has been standardised. However, the authors of many scientific papers [40,41,42,43] evaluating the shear design methods described in the above-named standards have stated that there were significant differences between the experimental and computed values. Therefore, the next objective of this research work is the evaluation of the shear design fibre-reinforced elements, based on the Model Code 2010 [17] and RILEM TC-162-TDF [39] standards, in terms of the possibility to use the methods for shear design of SFRWSC elements subject to bending. It should be noted that by using waste aggregate as a full-value construction material for the production of the tested SFRWSC, these studies are in line with the global trends related to sustainable development of the environment.

## 2. Test Materials

**Figure 1:**
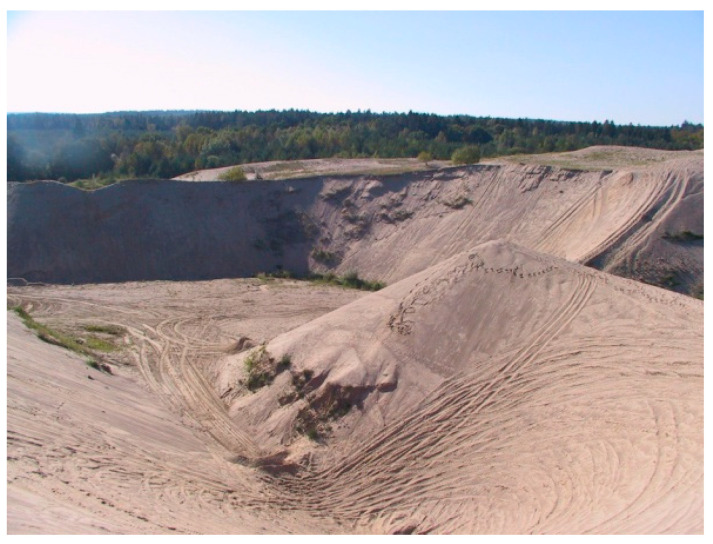
View of the sand heaps after the hydroclassification process in Pomerania (northern Poland). Reproduced with permission from ref. [13] published by Middle Pomeranian Scientific Society of the Environment Protection, 2015.

The reinforced concrete beams to be tested for shear capacity were made of SFRWSC containing 1.2% steel fibres, in relation to the volume of composite material [44].SFRWSC is a novel structural material, in which the used aggregate is a post-production waste. In the analysed composite, the used aggregate was sand of 4 mm granularity, which is a waste material of aggregate mines located in northern Poland (the Pomeranian region). In this area, a significant part of the output is subjected to the process of hydroclassification, which results in 80% sand and only 20% coarse aggregate. This disproportion leads to the situation where most of sand remains unused, in numerous dumps located near the aggregate mines (Figure 1).

**Figure 2:**
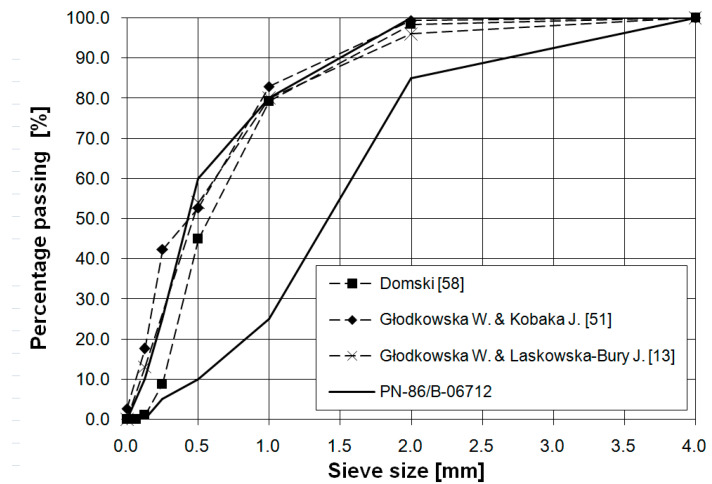
Grading curve of used aggregate and grading curves of other Pomeranianaggregates used in different research programs [13,51,58]. Reproduced with permission from ref. [52]; published by Middle Pomeranian Scientific Society of the Environment Protection, 2017.

The postulate to somehow utilise remaining waste sand dumps constitutes a world-wide tendency, consistent with Sustainable Ecological Development [45,46,47,48,49]. Similar phenomena of excessive sand fractions can be observed in other parts of the world, such as the Middle East or in North Africa [50]. Figure 2 presents waste sand grading curves appointed by various authors. These curves only insignificantly differ from one another, despite the fact that the sand used in these studies originated from various aggregate mines located in northern Poland. This indicates that all these deposits are post-glacial or fluvioglacial residues, developed in the same period [12,51,52].

The used fine aggregate complied with the requirements formulated for mineral aggregates recommended for ordinary concrete manufacturing. The content of mineral dust in the aggregate was below 3%, which allowed for it to be classified in the *f_3_* category, based on the PN-EN 12620 standard [53].

**Figure 3:**
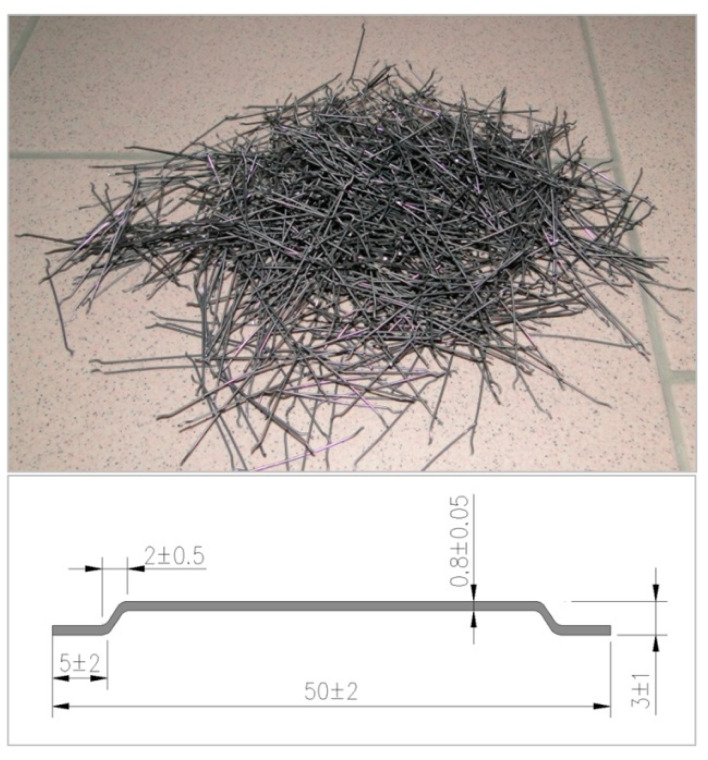
Fibre dimensions and close-up look.

Sand obtained from the hydroclassification process performed in the Mineral Raw Materials Mine in Podwilcze, Białogard Commune, was used (1570 kg/m^3^), together with Portland cement CEM II/A-V 42,5R (420 kg/m^3^), silica dust (21 kg/m^3^), superplasticiser FM series [54] (16.8 kg/m^3^),and tap water (160 kg/m^3^), in order to form the test elements. The fibre reinforcement comprised steel hook-end fibres (Figure 3) in the amount of 1.2% (94 kg/m^3^) and with *l/d* ratio *λ* = *l/d* = 62.5 (*l* = 50mm, *d* = 0.8mm) [55].The steel fibres had ITB technical approval No. AT-15-295/1999 [56], in accordance with the PN-EN14889-1 standard [57].

The fine aggregate composite matrix was designed through application of an analytical and experimental method. Modification of its composition by addition of silica dust and superplasticizer allowed us to obtain a*w/c* = 0.38 ratio. The fibres were placed in the composite mix at random. The technical characteristics of the steel fibres used in the tests are presented in Table 1.

The conditions of SFRWSC composing, care, and testing have been broadly described previously [11,12,13,51]. The mechano-physical properties of the analysed fibre composite with ordinary concrete are detailed below.

When considering fibrecomposite structural elements, one should first of all take into account the method of their design. For a designer of building structures, a standard-defined property is necessary, which determines the material’s ability to transmit tensile stresses after cracking, as provided by the manufacturer. Over the past 20 years, several proposals for a quantitative description of the behavior of cracked fiber-reinforced concrete under tensile conditions have been developed. The most common method for describing this property is given in the RILEM TC-162-TDF [39] recommendations. This method has been included in the European standard EN 14651 [16] and in the Model Code 2010 [17]. It consists of an experimental measurement of the relationship between the crack width (CMOD) and the load force on the bar in the middle of its span. The CMOD–Force relation determined as a result is used to define the so-called residual strengths: *f*_*R*,1_, *f*_*R*,2_, *f*_*R*,3_, and *f*_*R*,4_. The values of the residual strengths obtained in our tests are presented in Table 2. They denote the values of the tensile stresses in the cross-section for a given width of the CMOD crack, equal to 0.5, 1.5, 2.5, and 3.5 mm, respectively. The values of these strengths serve as the basis for the dimensioning of structural elements.

**Figure 4:**
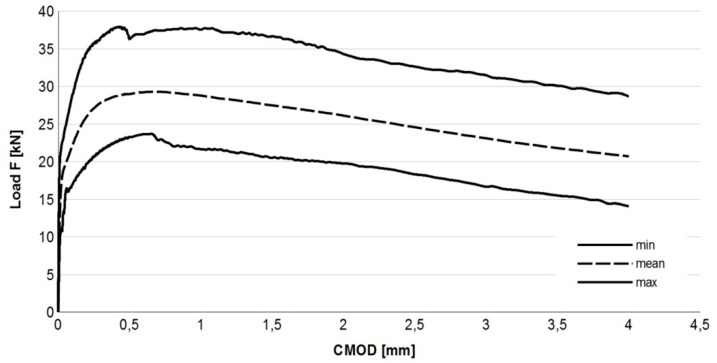
Load—CMOD relation for SFRWSC. Reproduced with permission from ref. [59]; published by Middle Pomeranian Scientific Society of the Environment Protection, 2015.

The Load– CMOD diagram resulting from residual strength test is depicted in Figure 4.

For easier interpretation of the test results, the graph boundaries (solid lines) and the mean force dependence on CMOD (dotted line line) are shown. The diagram shows a decrease in the destructive force as the CMOD value increases after the appearance of the first crack. The shape of the graph in Figure 4 indicates that the tested fibrocomposite shows the post-crack softening (pcs) feature. The obtained results clearly indicate the ductile nature of the SFRWSC with 1.2% fiber content. In accordance with the guidelines of the Model Code 2010 standard [17], the class of the tested SFRWSC was designated as 7b. It should be noted that the obtained values of the coefficient of variation (*ν*) given in Table 2, unlike the *ν* indices for the other properties of this material, are large. Unfortunately, tests of residual strength carried out with the use of beam elements are usually burdened with a large spread, amounting to an average of 20% [68] due to small bending areas in the beams, which has been confirmed in [69], among other works. Residual strength tests and their results have been discussed, in more detail, in [12,59].

It can be seen from Table 2 that SFRWSC with 1.2% steel fibre content has better or similar properties as ordinary concrete. The properties of this composite comply with the requirements formulated for structural materials; therefore, it may serve as a substitute for ordinary concrete. Partial substitution of concrete by the proposed fine aggregate composite with fibre reinforcement, featuring the same or better properties, provides a perfect solution for those regions that are short of natural coarse aggregate deposits. This will allow for regional aggregates to be used in a sustainable manner. Such actions will also contribute to the gradual depletion of sand dumps (Figure 1).

## 3. Methodology of Research and Test Elements

**Figure 5:**
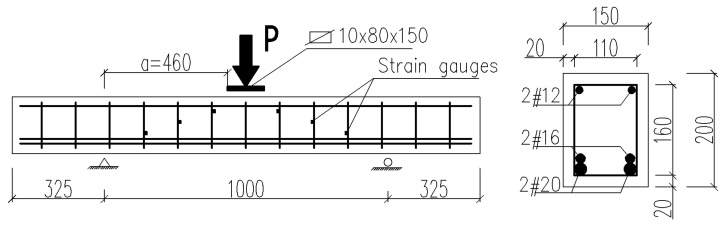
Specification of tested beams.

The test elements used in the shear capacity test are described in Figure 5 and Table 3. Additionally, test elements in form of cylinders (150 × 300 mm) and beams (150 × 150 × 700 mm) were made, in order to determine the fundamental properties of the proposed SFRWSC (Table 3).

**Figure 6:**
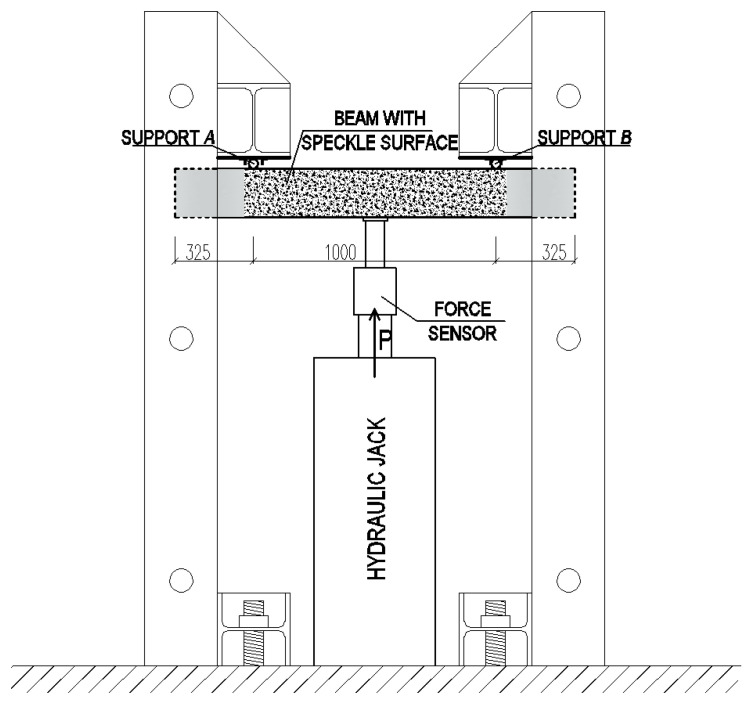
Schematic of experimental setup [44].

**Figure 7:**
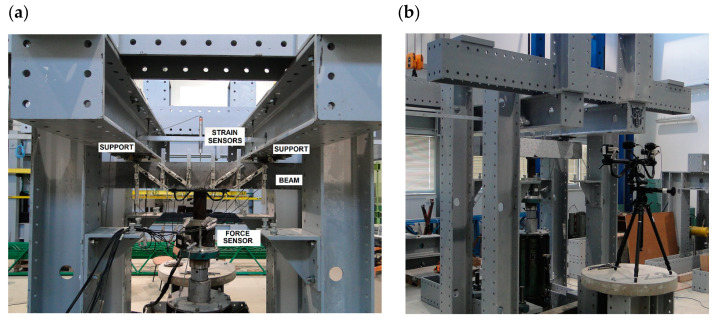
Stand for shear capacity testing of bend elements: (**a**) Beam side surface tested using SAD-256 system; and (**b**) beam side surface tested using Aramis 4M system [44].

In order to ensure shear failure a relatively large number of rebars was used in the tension face (2#20 and 2#16. In order to avoid the arch effect and the significant impact of the longitudinal reinforcement on the shearing force, shearing section *a* (Figure 5) was determined in such a way that the shear *a/d* ratio was about 3. In B and BF series beams, reinforcement was intentionally located in the compressed area (2#12), due to its significant impact on the shear capacity. Such prepared beams were stored, until tested, for 28 days at 20 ± 2 °C temperature and 100% relative humidity conditions. After 30 days, the beams were loaded. The stand used for shear capacity testing of SFRWSC beams is presented in Figure 6 and Figure 7.

**Figure 8:**
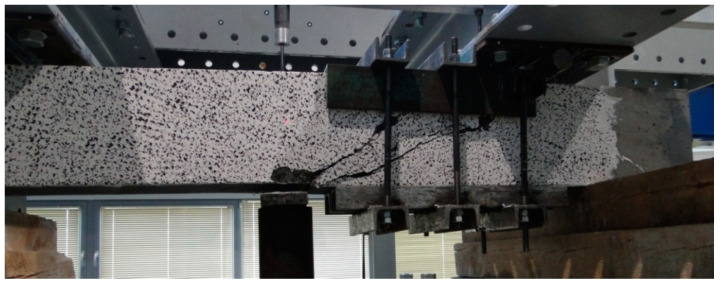
Beam reinforced with steel corset after the first testing stage.

The beams were tested using specially designed experimental setup, in the configuration of a reversed freely supported beam (Figure 6). The beams were loaded at a constant speed of ~4 kN/min, until failure. Two measuring techniques were used in the test: The SAD-256 data acquisition system (APIG Ltd., Łódź, Poland) (Figure 7a) and the Aramis 4M system (GOM Ltd., Braunschweig, Germany) (Figure 7b). Measurements were performed periodically at 0.5 Hz frequency, from the moment of load application until beam destruction. The SAD-256 sensor arrangement used for the measurement of surface deformation of one beam side (Figure 7b) was designed in such a way that the recording of deformations in the diagonal crack area was possible. The width of the cracks that were diagonal and perpendicular to the element axis, deformations of the second beam side surface, and deflections were measured using the Aramis 4M software (GOM Ltd., Braunschweig, Germany). To measure deformations of shear reinforcement, strain gauges were used, which were glued to the vertical parts of stirrups before concreting. Six strain gauges were used for each beam (i.e., three for each shear area). The beam loading force was recorded by a force sensor, located over the hydraulic jack, with 0.66 mV/V sensitivity. The beam span (Table 3) was selected in such a way that the shear failure at the first or second support could be recorded by the Aramis 4M (GOM Ltd., Braunschweig, Germany). Considering the adopted static arrangement, beam shear failure could occur within the first or second support area. For this reason, the beams were tested in two stages. In the first stage, the beam was subjected to loading until shear failure occurred. Then, the test was stopped, and the beam load reset. A steel corset, made of steel sections pulled into place by bolts (Figure 8), was put onto the failed shear area. The corset was intended to resist transversal forces in the fractured shear area in the second stage of the test. The beams with the steel corset were loaded until the second shear area failed.

## 4. Test Results and Their Analysis

### 4.1. Shear Behaviour of Bending Infibre-Reinforced Concrete Beams

Having analysed the beam deformation maps obtained from the Aramis 4M system for various levels of loading, five stages of functioning under shear conditions could be distinguished for the SFRWSC.

**Figure 9:**
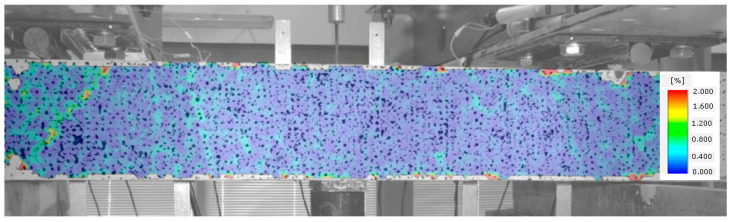
Stage I of shear behavior of element—no cracks.

When no cracks occur in a given element, longitudinal deformations of steel and fibre composite are the same. The element functions in the flexural phase until the tensile strength of the fibre composite is attained, as illustrated in Figure 9.

**Figure 10:**
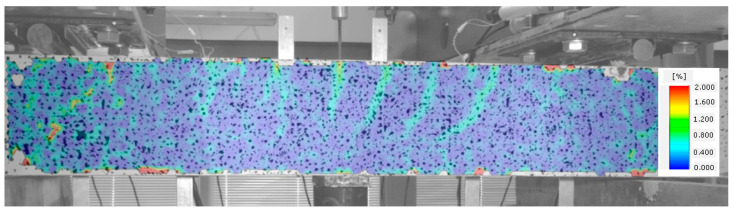
Stage II of shear behavior of element—flexural cracks have occurred.

Once the fibre composite tensile strength is exceeded, which corresponds to the occurrence of cracks, the beam starts functioning in stage II, as a cracked element. Cracks perpendicular to the element axis appear in the middle of its span. The beam functions in this phase until the diagonal tensile strength of the composite in the shear area is reached (Figure 10).

**Figure 11:**
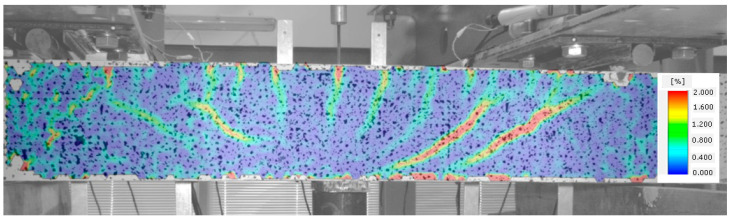
Stage III of shear behavior of element—occurrence of diagonal cracks in both shear areas.

In Figure 11, the occurrence of several diagonal cracks between the tensioned reinforcement and the compressed area of the cross-section of the analysed beam is already visible. At the same time, the direction of perpendicular cracks changes, slanting from perpendicular to diagonal position.

**Figure 12:**
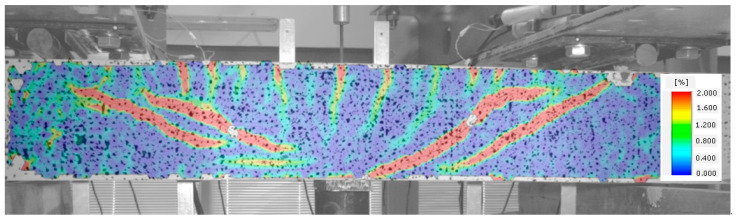
Stage IV of shear behavior of element—stabilization of the diagonal crack and arriving at the shear capacity.

A subsequent increase in beam loading results in increased crack width in the existing cracks, which means that the element is now in stage IV. In the case of beams with strong shear reinforcement, subsequent diagonal cracks may occur at this stage. The diagonal cracks become longer and approach the main reinforcement area. In stage IV, a critical diagonal crack may also appear, depending on the element shear capacity. Reaching of the shear capacity is shown in Figure 12.

**Figure 13:**
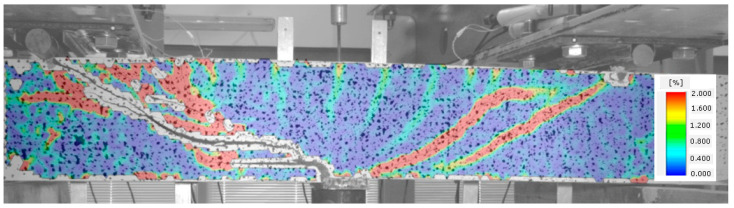
Stage V of shear behavior of element—shear compression failure of the beam.

Stage V is the stage of destruction of the element under shear force, through arriving at the composite diagonal compression strength (Figure 13). This type of destruction has been described, in [70], as a shear compression failure. It is observed in those beams that have strong main reinforcement with the simultaneous absence of or very poor transversal reinforcement. The cause of failure is a split fracture of the composite structure in the so-called compressed area (i.e., above the diagonal crack end), where a sort of pivot appears to occur.

**Figure 14:**
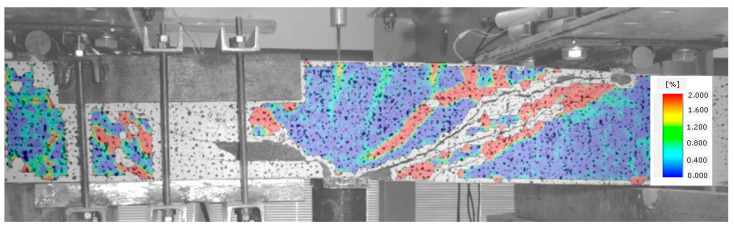
Stage V of shear behavior of element—shear tension failure of the beam.

Beam failure, due to loss of adhesion of the fibre composite to the reinforcement originating from elongation of the longitudinal crack at the main reinforcement height, is illustrated in Figure 14.

**Figure 15:**
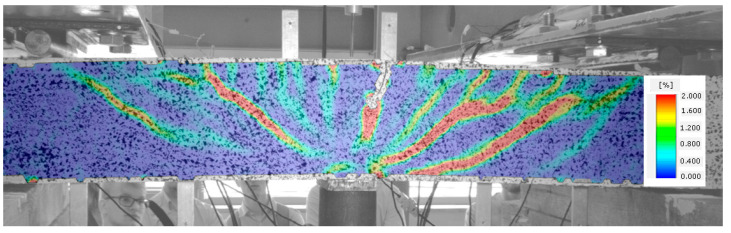
Stage V of shear behavior of element—element bending failure through yielding of the tensioned reinforcement.

Beams with strong shear reinforcement (BFSb series) were destroyed due to their arriving at the yield point of the tensioned reinforcement (Figure 15). The load shear capacity of an element is decided by its most strained and/or weakest cross-section. However, cracks originating in various element cross-sections are very important for its deformation, as they have an impact on rigidity of the entire element.

**Figure 16:**
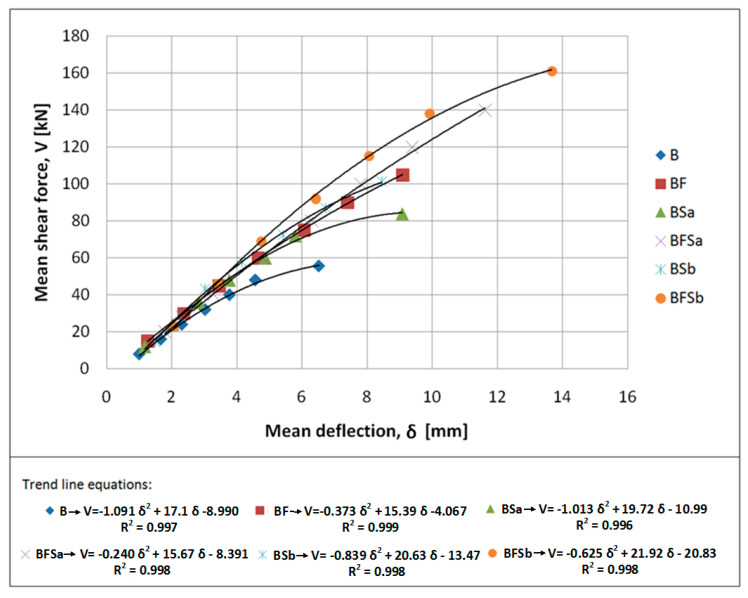
Relation between mean shear force and mean deflection for tested beams.

The application of steel fibres led to the failure images of SFRWSC beams shown above having a more ductile character, compared to fine aggregate cement composite beams without fibre reinforcement. The shear force–deflection relation, in the first stage of the test (pt. 3), is illustrated in Figure 16.

An analysis of Figure 16 clearly indicates the influence of steel fibers on the bearing capacity of the beams made of fine-aggregate fibre-composite near the support zone, and on the nature of the work of the beams after the appearance of the first diagonal crack. In the B series beams (without stirrups and steel fibers), when the crack appeared, the loading force remained at the same level. The appearance of successive diagonal cracks resulted in temporary load drops. However, in the remaining beams with shear reinforcement (stirrups and steel fibers), after the appearance of a diagonal crack, the load continued to increase and no decrease in the loading force was observed at the time of appearance of subsequent cracks. Both the stirrups and steel fibers affect this behavior of the beams, where the steel fibers can “bridge” cracks by transferring tensile stresses. In addition, the addition of steel fibers reduced the brittle nature of the material more effectively than stirrups. Analyzing the slope of the force (V)–deflection (δ) curves shown in Figure 16, it can be concluded that the steel fibers affected the bending stiffness, but the impact was not significant; which has been confirmed, in [15], in the case of elements with a high degree of main reinforcement. Yoo [71] and Ashour [72] also came to similar conclusions. It should be emphasized that, despite the slight influence of the addition of steel fibers on the stiffness, the final values of deflections at the maximum transverse force were much greater for beams reinforced with stirrups and steel fibers (approx. 13 mm), compared to those without fibers and stirrups (approx. 7 mm). The deflection values for the BF (with fibres) and BSa (with stirrups) series elements were similar, amounting to approx. 10 mm.

### 4.2. Beam Failure Models and Experimentally Determined Shear Capacity

**Figure 17:**
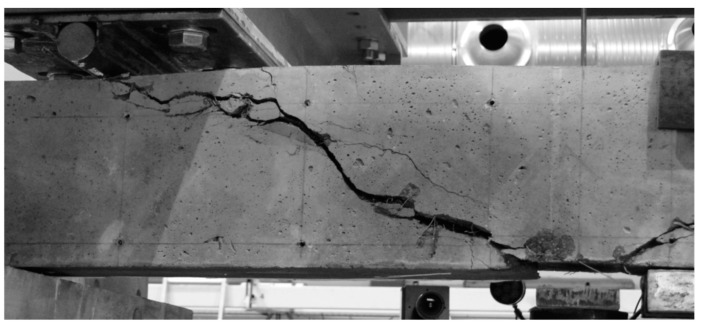
Example of a shear tension failure in the shear area of BF series beams.

Various failure models were observed during the tests, depending on the type of shear reinforcement and the combinations (Table 3) that were used [44]. The failure of B beams (without shear reinforcement) and beams having only fibre reinforcement (BF beams) had a shear tension character (Figure 17). Once the first diagonal cracks appeared, one increased its width as the load was increased. A number of small cracks occurred at the main reinforcement level, which means that gradual loss of the main reinforcement adhesion to the fibre composite occurred. Consequently, the depletion of shear capacity of such elements resulted from the main reinforcement steel slide at the place of anchoring on the support.

**Figure 18:**
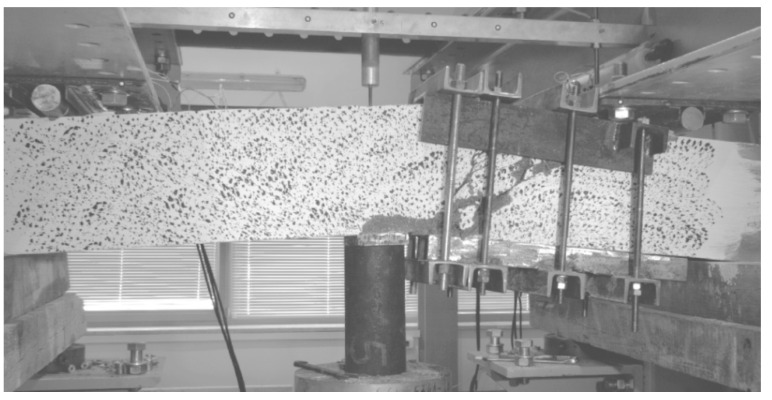
Typical secondary failure of a BFSa series beam.

**Figure 19:**
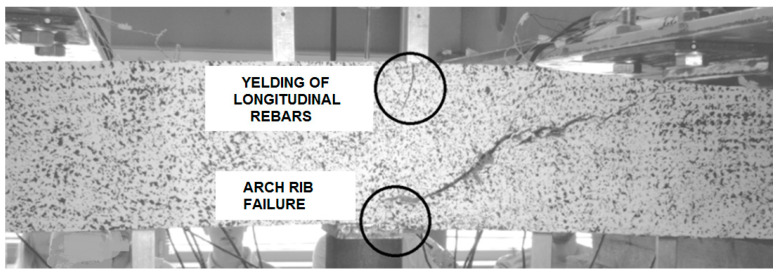
Example of a secondary BFSb series beams failure.

In the case of BFSa series beams (elements with 120 mm stirrup spacing), the failure was similar to that described above, but with the diagonal tension failure feature. Such a failure mode resulted from the high shear capacity of BSFa beams, and occurred in the case of beams with insufficient flexural reinforcement over their entire span. In this case, the flexural reinforcement steel yield point was observed not in the middle of the element span, but at the section of the simultaneous action of the bending moment and transversal force. This resulted in an increase in width of one of the diagonal cracks, leading to its elongation and penetration into the compressed cross-section area. This crack caused, in effect, the crashing of concrete in the cross-section above the crack. Therefore, for this beam series, the second test stage—which was aimed to define the shear capacity of the undamaged shear area—failed. In this particular case, the beams failed because of bending (Figure 18). In most cases of BFSa series beams (featuring greater stirrup spacing equal to 120 mm) and BSa series beams (with stirrup spacing of 120 mm, no steel fibres added), the stirrups through which the diagonal crack passed had fractured. In the destruction of BFSb series beams (stirrups spaced at 90 mm), which showed the highest shear capacity, failed due to yielding of the flexural reinforcement (flexural failure). Finally, due to high deformation, the compressed area of the element crashed. In effect, a secondary shear failure of the diagonal tension type occurred (Figure 19).

Table 4 shows the mean values of forces *V_cr_* (shearing force at which a diagonal crack occurs) and *V_ult_* (the ultimate shear force) obtained from tests for particular beam series.

Table 5 shows the effects of reinforcement of SFRWSC beams obtained in the tests.

The highest values of the shear force (*V_cr_*), for which the initial diagonal cracking has been observed, and the ultimate shear force (*V_ult_*) were observed for the beams reinforced with both stirrups and steel fibres. The reduction of stirrup spacing from 120 mm to 90 mm contributed to an increase in the shear capacity of elements by 23%, on average, for the beams without fibres, and 13% for the beams with fibres. The analysis of the results obtained for the beams reinforced only with stirrups and only with steel fibres led to the conclusion that slightly higher carrying capacities occurred in the case of the beams without stirrups. Compared to BFSa beams, the tested forces, *V_ult_*, were lower by 40% for BSa beams and by 26% for BF beams. Despite the fact that addition of the second type of shear reinforcement provided a lesser reinforcement effect (see Table 5), the impact of steel fibres and stirrups on the shear capacity added up, which originated from the results for BFSa and BFSb series beams. Similar conclusions have also been drawn by other authors, who analysed the co-operation of stirrups with steel fibres in resistance to transversal forces [21,22,23,73].Our research also showed that fibre reinforcement had an advantageous impact on the occurrence of the first diagonal crack, in comparison to those beams that contained no steel fibres. The value of the transversal cracking force (*V_cr_*) in the fibre composite elements was higher (by approximately 38%), compared to the beams without fibre reinforcement. In beams of BF, BSa, and BFSa series, the ratio of the cracking force (*V_cr_*) to the ultimate shearing force (*V_ult_*) was constant and amounted to approximately 0.56. The insignificant increase in *V_ult_*, compared to *V_cr_*, for B series beams originated from the fact that the shearing force was reduced by occurrence of the so-called “dowel action”, engaging the aggregate and the compressed area.

**Figure 20:**
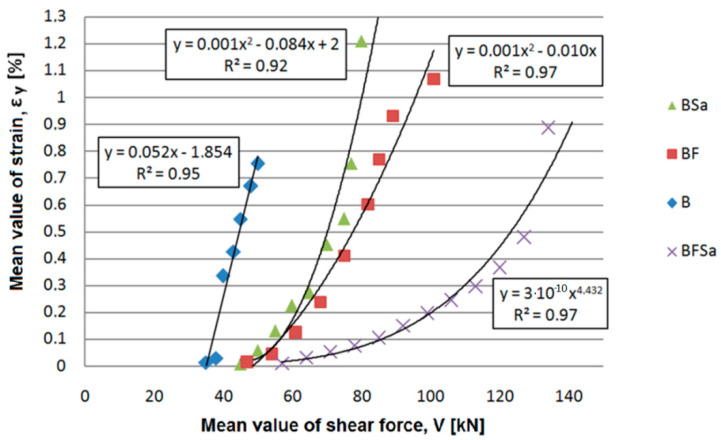
An example of dependence of the side surface strain at element height (*ε_y_*) on the transversal force (*V*) for B, BSa, and BF series beams at the first stage of the test.

Consequently, our research work demonstrated that the addition of steel fibres to SFRWSC had an enormous impact on the resistance to transversal forces. It increased the shear capacity by approximately 90% in beams with no stirrups and that by approximately 65% in beams with stirrups. The fibre functioning character in the shear area was more beneficial than stirrup action, due to more ductile character of the material. An example of dependence of the side surface strain at element height (*ε_y_*) on the transversal force (*V*) is shown in Figure 20.

We also ascertained that the impact of steel fibres and stirrups added up, in terms of resistance to transversal forces, in order to increase the element shear capacity. Furthermore, it was found that the concentration of stirrups had no significant impact on the cracking force (*V_cr_*), for the beams with fibres and without. Similar results have also been obtained by Lim and Oh [74]. We also found that, as the shear capacity of BF and BSb series beams was comparable, stirrups #4.5 spaced at 90 mm intervals reinforced the beams to the same degree as the content of steel fibres amounting to 94 kg/m^3^ (1.2%).

### 4.3. Diagonal Cracks

**Figure 21:**
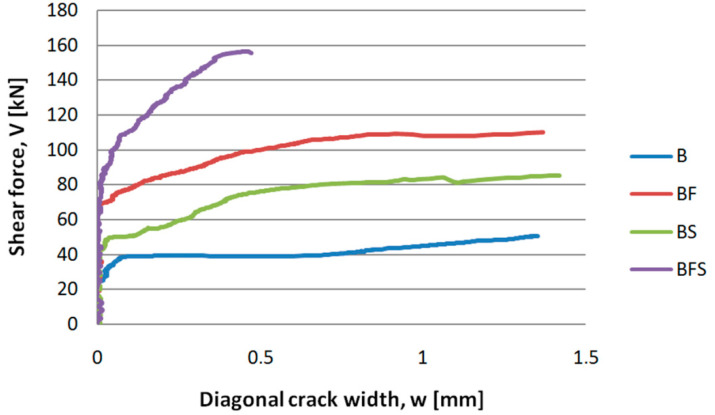
Shear force (*V*) versus diagonal cracks opening width (*w*) for selected B, BF, BS, and BFS series beams.

The dependence of the shear force (*V*) on the width of the diagonal crack opening (*w*) in beams made of SFRWSC is shown in Figure 21. For elements reinforced with fibres (BF series) a higher cracking force than that in beams reinforced with stirrups (BS series) was noted, which may have a beneficial impact, in terms of reduction of the shear span requiring shear reinforcement. Curves describing the dependence of the transversal force on the width of the diagonal crack opening for beams with fibre reinforcement (BF series) and reinforced only with stirrups (BS series) featured a similar angle of inclination to the horizontal axis. Such a course of the curves, as shown in Figure 16, indicates the similar functions of steel fibres and stirrups after element cracking. At the same time, in the case of BFS series beams (reinforced with stirrups and steel fibres), the effects of the fibres and stirrups add up, as indicated by the slow increase in the crack opening width (*w*) with increasing transversal force value (*V*). It should be noted that the maximum width of the diagonal crack opening in BFS-type elements was significantly lower than that observed in BF and BS series beams. This resulted in a higher number of cracks in BFS-type elements, where the shear reinforcement was provided by stirrups and steel fibres. Ultimately, it can be stated that fibres have strong impact, not only on the shear capacity of SFRWSC elements, but also on their diagonal cracking. Comparing the test results obtained for BF and BS series beams, it can be stated that the same values of crack widths occurred at higher transversal force values (by approximately 40%) for the beams containing fibres. It should be noted that the *V-w* curves for BF and BS elements featured a similar angle of inclination to the horizontal axis, which may indicate a similar mechanism of resistance to transversal force after cracking by stirrups and steel fibres.

The highest number of cracks for one shear area was observed in beams reinforced with stirrups and fibres: either 3 or 4. For beams with only fibres, the number of cracks was similar to that for beams with only stirrups: 2 or 3, on average. The presence of greater number of cracks simultaneously resulted in smaller crack widths. For beams with stirrups and fibres, the opening width of diagonal cracks did not exceed 1 mm, while that for beams with only fibres was, on average, 1.25 mm.

In summary, in terms of diagonal cracking, the elements containing steel fibres and stirrups behaved best, as an increase in force caused a considerably lower increment of crack opening width, where as a constant value was observed in the case of beams reinforced with fibres (BF) or stirrups (BS).

## 5. Computational Analysis

The shear capacity of beams made of SFRWSC was computed through the application of two methods—those of RILEM TC-162-TDF [39] and Model Code 2010—using the SMCFT [17] method for the second approximation levels, as well as the former method. The objective of this computation was to prove the applicability of European standards for shear design of fibre concrete cross-sections; that is, to verify whether Model Code and RILEM can be used in the shear design of SFRWSC cross-sections.

Average values of features of SFRWSC with and without steel fibres, as well as average values of features of reinforcing steel, were used in the computations (Table 2 and Table 3). Values of the axial tensile strength of SFRWSC were determined using the relationship of Amin and Foster [75], which allows for the transformation of residual strengths to axial tensile strengths. The angle of inclination of compression struts (*θ*) for the SMCFT [17] method was assumed as the minimum; whereas, for the two other methods (RILEM and Model Code 2010—former method), it was *θ* = 30°. Safety coefficients in the shear capacity computations were as follows: *γ_f_ = γ_c_ =* 1.0.

To determine the shear capacity of BSa and BSb series beams through application of the SMCFT method, the second level of approximation was used for elements containing steel fibres, with the method for computation of the coefficient *k_v_* taking into account the share of the maximum aggregate grain [17]. To determine the total shear capacity (*V_Rd_*), notation for the third level of approximation was used, taking into account the influence of the cross-sectional shear capacity in the element without transversal reinforcement(*V_Rd,c_*), as well as the shear capacity due to transversal reinforcement (*V_Rd,s_*).

**Figure 22:**
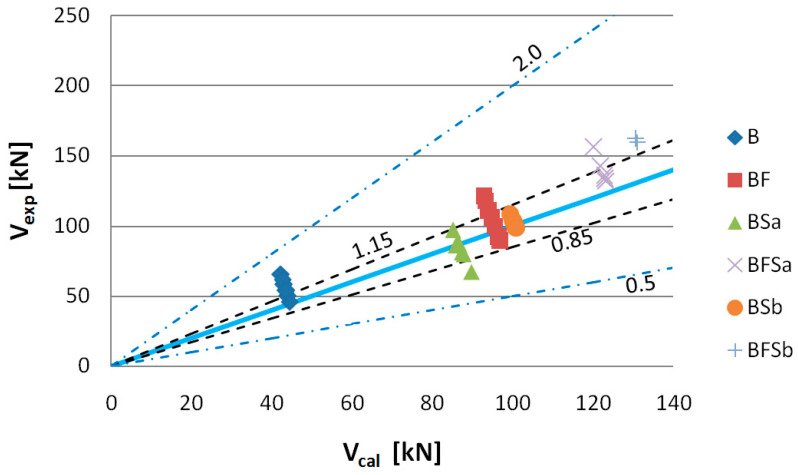
Values of experimentally fixed (*V_exp_*) versus computational (*V_cal_*) shear capacity determined according to Model Code using the SMCFT method (*θ* = min *θ*).

**Figure 23:**
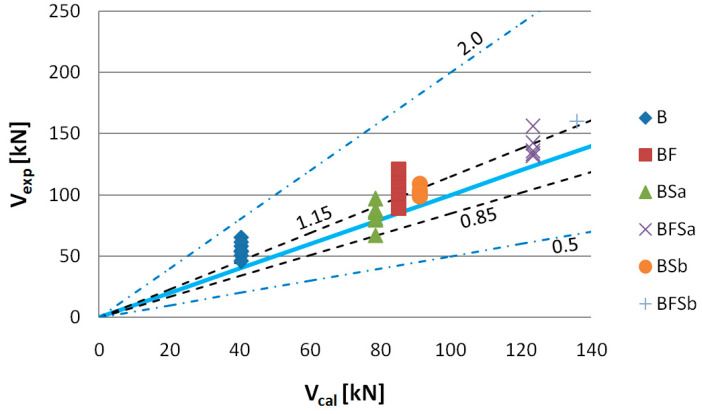
Values of experimentally fixed (*V_exp_*) versus computational (*V_cal_*) shear capacity determined according to the former method Model Code 2010 (*θ* = 30°).

**Figure 24:**
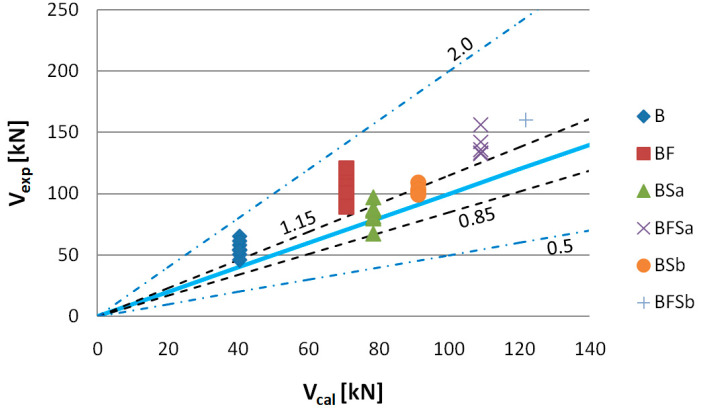
Values of experimentally fixed (*V_exp_*) versus computational (*V_cal_*) shear capacity determined according to the RILEM TC-162-TDF method (*θ* = 30°).

**Figure 25:**
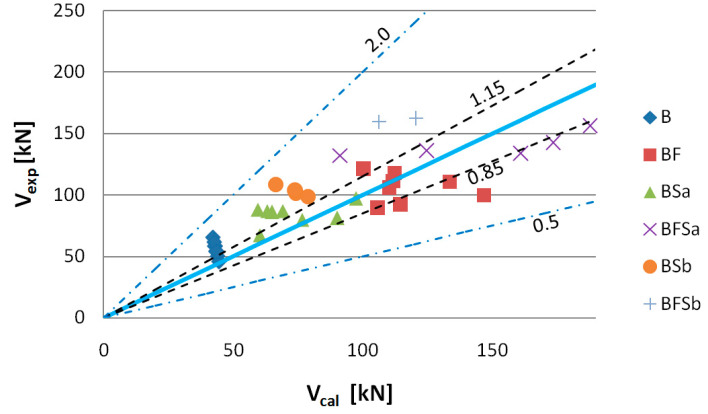
Values of experimentally fixed (*V_exp_*) versus computational (*V_cal_*) shear capacity determined according to the former Model Code 2010 SMCFT method (measured angle *θ*).

**Figure 26:**
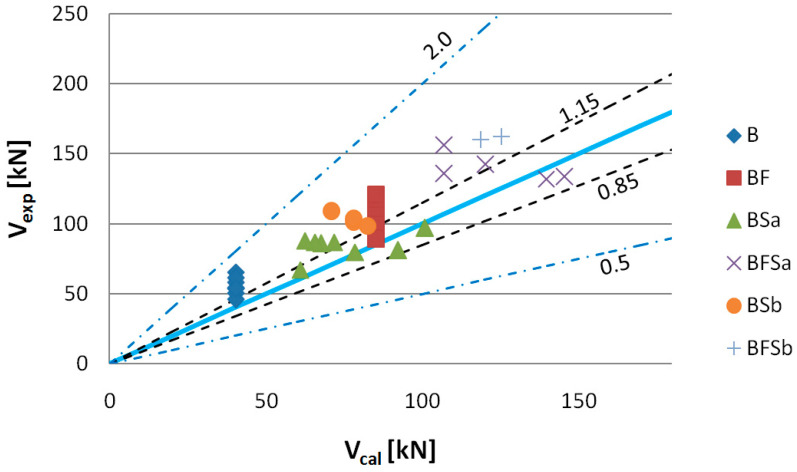
Values of experimentally fixed (*V_exp_*) versus computational (*V_cal_*) shear capacity determined according to Model Code 2010 (measured angle *θ*).

**Figure 27:**
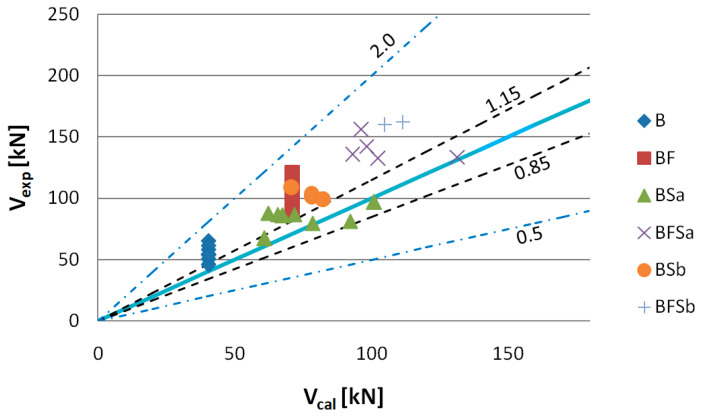
Values of experimentally fixed (*V_exp_*) versus computational (*V_cal_*) shear capacity determined according to RILEM TC-162-TDF (measured angle *θ*).

To assess the usability of the RILEMTC-162-TDF and Model Code 2010 methods to determine the shear capacity of elements made of SFRWSC, the criterion of Baghi and Barosso [76] was used. The comparative quantity in this criterion is the ratio of the value of the experimentally fixed shear capacities (*V_exp_*) to computational values (*V_cal_*). The shear capacity assessment criteria are shown in Table 6 and Figure 22, Figure 23, Figure 24, Figure 25, Figure 26 and Figure 27.

The experimentally determined shear capacity values (*V_exp_*) are set, in Figure 22, together with the analytically determined (*V_cal_*) values based on the FIB Model Code 2010 using the SMCFT method. After analysis of the graph, it can be stated that the theoretical shear capacity values for B and BFSb series beams are lower than those which were determined experimentally. The computational shear capacity values for B series beams at 75%, as well as those for BFSb beams at 100%, fit into the “conservative” classification interval (Table 6). The best compatibility with the *V_exp_* = *V_cal_* straight line occurred for BSa and BSb series elements, for which the percentages of results complying with the “appropriate safety” criterion were 88% and 100%, respectively. The computational shear capacities for BF and BFSa series beams were classified as 50% within the 0.85–1.15 interval and 50% within the 1.15–2.0 interval.

Figure 23 shows values of shear capacity *V_exp_* to *V_cal_*, computed according to the former Model Code method. The results obtained show that the shear capacities for B series beams were much lower than the experimental shear capacity values. It appears, from our analysis, that 88% of the shear capacity results should be treated—in accordance with the adopted criterion—as “conservative”. The values of shear capacity calculated for BSa and BSb series beams were close to those determined by application of the SMCFT method. In this case, the shear capacity values were qualified as being “appropriate safety”. The highest discrepancies were observed for the shear capacity values of BF series beams calculated by application of the SMCFT method. A total of 75% of beam shear capacity values were classified within the 1.15–2.0 interval (Table 6). Having applied the former Model Code method, it is clear that the impact of steel fibres on the shear capacity of SFRWSC beams is lower.

Figure 24 shows the values of shear capacity *V_exp_* to *V_cal_*, computed according to the RILEM TC-162-TDF method. Having analysed the shear capacity computation results, we have found that, when using this method, the highest discrepancies between the computational and experimental shear capacity values were obtained, where the highest discrepancy was observed for BF series beams. In this case, 100% of the shear capacity values were classified as “conservative”. The impact of steel fibres on shear capacity fell into the1.15–2.0 interval (Table 6) for BFSa and BFSb series beams, making them also less effective. The computational shear capacity values, determined by application of the former Model Code method with adoption of the same shear capacity computational algorithm as in EC-2 [77], for elements containing no shear reinforcement and all beams with stirrups, overlapped. It should also be pointed out that Figure 25, Figure 26 and Figure 27 show shear capacity values for BFSb series beams, despite their bending failure; however, assuming that shear force acting together with the destructive momentum is the minimum experimentally fixed shear capacity (this has been indicated by stirrups functioning in BFSb beams, as described below), it can be stated that the highest *V_exp_* to *V_cal_* differences occurred with the RILEM method (difference min. 33%), compared to the former Model Code method (min. 20%) and the SMCFT method (min. 5%). Large differences in shearing capacity values obtained by application of the RILEMTC-162-TDF method have also been confirmed by other authors, such as Matthys [78], Parmentier [41], and Arslan [79,80].

Figure 25, Figure 26 and Figure 27 show comparisons of the experimentally fixed shear capacity values for SFRWSC beams with the values computed using the RILEM TC-162-TDF method [39], Model Code 2010 [17] using the SMCFT method, and the former method. The adoption of real (obtained from tests) angles *θ* caused a significant computational discrepancy in the shear capacity values. The SMCFT method (Figure 25) requires particular attention; in this method, the angle *θ* is used not only for computation of the shear capacity of beams with stirrups, but also in the elements containing only fibres. In this case, the majority of shear capacity values computed for BF series beams were higher than the shearing capacity values found experimentally, causing an opposite situation than if the minimum value of angle *θ* was adopted. Three of the determined shear capacity values qualified as “dangerous” (Table 6). A similar situation occurred in the case of BSa series beams, when using the RILEM and former Model Code methods. In the remaining cases, particularly for extremely high values of angle *θ*, the computational values of shear capacity were higher (even by 50%).

For better interpretation purposes, and for assessment of the computed shear capacity values determined using the minimum and measured angle *θ*, the Integral Absolute Error (*IAE*) index was used. Having analysed *IAE* indices for *θ = min* and *θ* = 30°, the best compatibility was observed for BSa and BSb series beams, particularly in the case of the SMCFT method. In this case, the *IAE* index value did not exceed 10%. For BF series beams, the SMCFT method also showed the best compatibility (equal to 15%). The worst compatibility (exceeding 30%)was obtained with the RILEMTC-162-TDF method. *IAE* indices for BFSa and BFSb series beams had the lowest values with the Model Code 2010 method (12% and 16%, respectively); whereas, with the RILEM method, they exceeded 20%. The RILEMTC-162-TDF method, when analysed by other authors [78,79,80], also indicated significant discrepancies, with respect to computed values.

**Figure 28:**
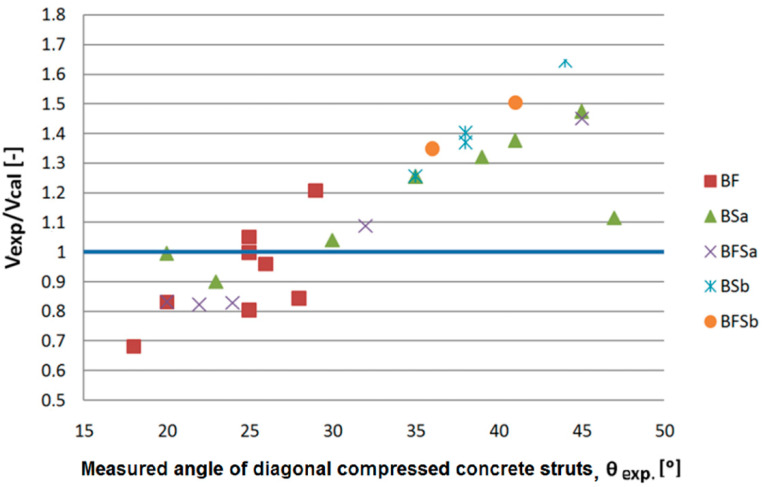
*V_exp_/V_cal_* values versus values of the measured angle of compression struts inclination (exp. *θ*) obtained in the tests.

The IAEs for the measured angle *θ* criterion were higher, which can also be confirmed through observation of Figure 25, Figure 26 and Figure 27. A conclusion can be drawn here: the adoption of a proper angle of inclination for the concrete and SFRWSC compression struts constitutes a key aspect in the determination of shear capacity. A large spread of measured angles *θ* resulted in higher *IAE* index values. To illustrate the impact of angle *θ* on the computational shear capacity values, values of *V_exp_*/*V_cal_* were analysed, comparing them with the compression strut inclination angles observed during testing (Figure 28). In this analysis, only the SMCFT method was considered, in which the angle *θ* (apart from the stirrup shear capacity) has an impact on the shear capacity of the fibres alone. From Figure 25, it is apparent that, with an increase in the compression strut inclination angle, the shear capacity values decreased. The highest values of *V_exp_*/*V_cal_* occurred for angles *θ* exceeding 35°, where the computational shear capacity value was lower than the experimentally fixed value by anywhere from 25% to over 50%. For the lowest angles *θ*, the situation was opposite, and the theoretical shear capacity values were exorbitant. However, in this case, the maximum difference was approximately 30%. A conclusion can be drawn here: assuming the tested SFRWSC, computation using high angle *θ* values results in considerable underestimation of the shear capacity *V_Rd,s_*, even in the event of an actual occurrence of such an angle. This has happened to appear in all beams with a high degree of shear reinforcement (BSb and BFSb series beams). The best compatibility (*V_exp_*/*V_cal_* = 1) was obtained when the angle fell within the interval of 20° to 32° for BF, BSb, and BFSa series beams. At this point, it should be noted that, in the case of beams containing only fibres, the best compatibility was achieved when the real angle was equal to 25°. This is a very important conclusion because, according to the analytical method proposed by the Model Code, the minimum angle of inclination of compression struts calculated for those beams was approximately 33°. The tests and computations performed further indicate a necessity to correct the procedure of computation for the minimum angle inclination value for compression struts, according to the SMCFT method, for the tested SFRWSC. This was indicated through analysis of the real and minimum angles *θ*, as well as the impact of the real angle *θ* on the experimentally fixed and computed shear capacity values for SFRWSC (Figure 28).

## 6. Conclusions

Experimental testing and computation of the shear capacity of reinforced concrete elements, made using a novel fine aggregate composite with and without steel fibres, allowed us to formulate the following conclusions:

(1). The fine aggregate concrete composite without steel fibres behaves like ordinary concrete, as a typical flexural element with conventional steel rebars. The computation results for the shear capacity of such elements using the RILEM and Model Code 2010 methods are fully satisfactory.
(2). According to the ultimate limit stage of shear capacity, fibre reinforcement in SFRWSC beams considerably contributes to the resistance of shearing forces and increases the shearing capacity (amounting to approximately 80%, compared to elements without shear reinforcement).
(3). The functional character of steel fibres in the shear area is better than that of stirrups, due to their more ductile material character. This conclusion was confirmed by the dependence of the transversal force (*V*) on deformations (*ε*), determined at the side beam surface at its height. The effects of steel fibres and stirrups add up, in terms of resistance to transversal forces, both in the aspect of increasing the shear capacity, as well the element deformability.
(4). Steel fibres at an amount of 1.2% in SFRWSC affect the occurrence of diagonal cracks. Diagonal cracks in beams with fibres appeared at higher transversal force values than in the case of elements without fibres. The diagonal crack analysis showed that the shear crack force was approximately 57% of the ultimate shear force. Thus, the steel fibres contribute not only to increasing the shear capacity, but also to the increase inthe shear crack force.
(5). The addition of 1.2% steel fibres considerably improves the shear capacity of SFRWSC elements. This allows for the reduction of conventional reinforcement in such elements. Due to the high residual tensile strength (*f_Ftu_*) of fibre-reinforced concrete, the analysed SFRWSC does not require minimum reinforcement in the form of stirrups, as indicated by the following formula:

(1) $$f_{Ftu}=2.18\;\mathrm{MPa}>0.08\cdot \sqrt{f_{c}}=0.6\;\mathrm{MPa}$$

(6). With regard to elements made of SFRWSC, the shear capacity values computed using the RILEM and Model Code 2010 methods were higher than the experimentally determined values. The obtained results indicate a necessity for correction of these methods, in order to apply them to the shear design of elements made of SFRWSC. Other researches who have been working on classical fibre-reinforced concrete have arrived at similar conclusions. Furthermore, the computation procedure using the minimum angle of inclination of compression struts, according to the SMCFT method, in terms of the tested fibre-reinforced composite, requires correction, due to great differences between the measured and computed angle values. What the authors consider as the further direction of research and analysis.
(7). Considering the mechano-physical properties of SFRWSC and the shear capacity test results presented in this paper, as well as the flexural capacity values for beams made of this fibre-reinforced composite, as described in [15,36], the assumption can be made that this material can successfully be used as a structural material. The developed SFRWSC, the properties of which comply with the requirements set for structural materials, could be used as an alternative solution for ordinary concrete in some applications, providing an opportunity to utilise the waste sands piled in Pomerania (Poland), in the Middle East, or in North Africa.

The conclusions presented in the article should not be generalized. Experimental tests and calculations were carried out for an innovative SFRWSC with a grain size of up to 2 mm, a specific cross-section of beams, the amount of longitudinal and transverse reinforcement and a static scheme. The obtained results of the research and the conducted literature studies in the field of the subject of the presented work allowed for the formulation of further research directions. The use of numerical modeling to predict the behavior of beams with different static patterns, for different types of element cross-sections, values of classical and fiber reinforcement, variable *a*/*d* shear slenderness, or various actions and their combinations is one of them. Thus, the analyzes will provide a large number of results that cannot be registered in an experiment with a measuring apparatus. Moreover, numerical analyzes will allow to identify the necessary areas for further experimental research and to introduce changes in the experimental program in order to improve the applied model. Due to the highly non-linear nature of the analyzed shear process in the fiber-reinforced concrete elements and the analysis until failure, it will be advantageous to use a quasi-static calculation strategy.

## Figures and Tables

**Table 1:** Technical characteristics of the steel fibres used in the tests ([55,56]).

Pos.	Feature	Value	UoM
1	Structural group	I	-
2	Tensile strength	1160 ± 7	N/mm^2^
3	Young’s modulus	210,000	N/mm^2^
4	Consistency by fibre content 14–15 kg/m^3^, measured by application of Ve-be method	4	s
5	Impact on concrete strength at CMOD * = 0.5 mm	1.5	N/mm^2^
6	Impact on concrete strength at CMOD * = 3.5 mm	1.0	N/mm^2^

*—cut crack width according to PN-EN 14651 standard method [16].

**Table 2:** Mechano-physical properties of the analysed fibre composite with ordinary concrete [12,14,51,59].

Property	Material		Methodology of Determination
	Fibrecomposite (with Coefficient of Variation)	Ordinaryconcrete	
Aparent density in dry condition: ρ, [kg/m^3^]:	2290 (*ν* = 0.7%)	2000–2600	PN-EN 12390-7 [60]
Compression strength: $f_{c\;cyl},$ [MPa]	64.4 (*ν* = 6%)	12–50 (PN-EN 1992-1-1)	PN-EN 12390-3 [61]
Compression strength: $f_{c\;cube,}$ [MPa]	67.6 (*ν* = 3%)	15–60 (PN-EN 1992-1-1)	
Split tensile strength: $f_{t\;\mathrm{spl}}$ [MPa]	7.3 (*ν* = 8%)	3.0–3.7	PN-EN 12390-6 [62]
Static modulus of elasticity: $E_{cm},$ [GPa]	36.7 (*ν* = 7%)	29–37	PN-EN 12390-13 [63]
Dynamic modulus of elasticity: *E_d_* [GPa]	45.9 (*ν* = 1%)	*E_cm_* = 0.83 *E_d_*	Neville A.M. [64]
Creep: $\varepsilon_{p},$ [‰]	0.26 (*ν* = 4%)	0.1–1.0	ITB 194/98 instructions [65]
Shrinkage: $\varepsilon_{cs},$ [‰]	0.88 (*ν* = 4%)	0.2–0.6	
Abrasion resistance: A, [cm^3^/50 cm^2^]	9.0 (*ν* = 7%)	1.5–22	PN-EN-13892-3 [66]
Residual strength: *f_R_*, [MPa]	$f_{R1}=9.3$ (*ν* = 13%)	Not applicable	PN-EN 14651 [16]
	$f_{R2}=8.8$ (*ν*= 15%)		
	$f_{R3}$= 7.9 (*ν* = 15%)		
	$f_{R4}$= 7.0 (*ν* = 17%)		
Shear strength: τ, [MPa]	12.9 (*ν* = 8%)	-	JCI-SF6 [67]

**Table 3:** Description of the test elements.

Beam Marking	Stirrups	Fibre
B (4 pcs.)	none	0%
BF (4 pcs.)	none	1.2%
BSa (4 pcs.)	#4.5 @ 120	0%
BSb (2 pcs.)	#4.5 @ 90	0%
BFSa (4pcs.)	#4.5 @ 120	1.2%
BF2b (2 pcs.)	#4.5 @ 90	1.2%
Composite properties: *f_c_* = 52.6 MPa, *f_ct_* = 3.3 MPa, *f_cf_*= 64.4 MPa, *f*_*R*1_ = 9.27 MPa, *f*_*R*2_ = 8.80 MPa, *f*_*R*3_ = 7.87 MPa, *f*_*R*4_ = 6.98 MPa, *E_cm_* = 36.7 MPa Steel properties: *f_y_* = 529 MPa, *f_t_* = 650 MPa, *E_s_* = 200 GPa, *f_yw_* = 584 MPa, *f_tw_* = 615 MPa		

**Table 4:** The mean values of forces *V_cr_* and *V_ult_* for particular beam series.

Beam	*V_cr_* [kN]	Standard Deviations [kN]	*V_ult_* [kN]	Standard Deviations [kN]	*V_cr_/V_ult_* [-]
B	44.28	16.42	55.57	19.40	0.80
BF	61.00	22.53	106.25	37.04	0.57
BSa	47.18	17.30	84.21	29.19	0.56
BFSa	79.93	31.31	140.27	57.92	0.57
BSb	45.29	7.08	103.32	4.27	0.43
BFSb	64.78	16.86	161.2 *	-	0.40

*—flexural failure.

**Table 5:** Increase coefficients for particular beam series.

Increase Coefficient						
Beam	*V_ult_/V_ult_^B^*	*V_ult_/V_ult_^BF^*	*V_ult_/V_ult_^BSa^*	*V_ult_/V_ult_^BFSa^*	*V_ult_/V_ult_^BSb^*	*V_ult_/V_ult_^BFSb^*
B	1.00	0.52	0.66	0.40	0.53	0.34
BF	1.91	1.00	1.26	0.76	1.03	0.66
BSa	1.51	0.79	1.00	0.60	0.82	0.52
BFSa	2.52	1.32	1.67	1.00	1.36	0.87
BSb	1.86	0.97	1.23	0.74	1.00	0.64
BFSb	>2.90	>1.52	>1.91	>1.13	>1.56	1.00

**Table 6:** The criteria for assessment of experimentally fixed (*V_exp_*) and computational (*V_cal_*) shear capacity values [76].

*V_exp_*/*V_cal_*	Classification
<0.5	Extremelydangerous
[0.5–0.85]	Dangerous
[0.85–1.15]	Appropriate Safety
[1.15–2.0]	Conservative
≥2.0	Extremely Conservative

## Data Availability

The data presented in this study are available on request from the corresponding author.

## Nomenclature

CMOD	Crack Mouth Opening Displacement
SFRWSC	Steel Fibre Reinforced Waste Sand Concrete
SMCFT	Simplified Modified Compression Field Theory
*Ecm*	static modulus of elasticity
*Ed*	dynamic modulus of elasticity
*Es*	modulus of elasticity of longitudinal steel rebars
*Gd*	dynamic modulus of rigidity
*Vcr*	shear crack force
*Vcal*	calculated shear capacity
*Vexp*	experimental shear capacity
*VRd*	shear capacity
*VRd,c*	specimen’s shear capacity without shear reinforcement
*VRd,s*	shear capacity increase due to conventional shear reinforcement
*Vult*	ultimate shear force
*δ*	deflection
*εy*	mean value of strain
*θ*	compression strut angle
*ν*	coefficient of variation
*a*	shear span,
*d*	effective depth of beam
*fc,f*	fibre-reinforced composite compression strength
*fc*	fibre-less composite compression strength
*fct*	fibre-less composite tensile strength
*fFtu*	residual tensile strength of fibre-reinforced concrete defined for wu = 1.5 mm
*fR,1 fR,2 fR,3 fR,4*	residual strengths determined in accordance with relevant standard for CMOD = 0.5 mm, 1.5 mm, 2.5 mm, and 3.5 mm
*fy*	longitudinal reinforcement steel yielding strength
*fyw*	shear reinforcement (stirrups) steel yielding strength
*ft,spl*	split tensile strength
*ft*	tensile strength of longitudinal steel rebars
*ftw*	tensile strength of shear reinforcement (stirrups)
*s*	standard deviation
*w*	crack width
